# Identifying cross-cultural variations in psychostimulant use for attention deficit hyperactivity disorder using linked data

**DOI:** 10.1186/s13034-017-0152-9

**Published:** 2017-03-20

**Authors:** Manonita Ghosh, C. D’Arcy J. Holman, David B. Preen

**Affiliations:** School of Population Health, The University of Western Australia, 35 Stirling Highway, Crawley, WA 6009 Australia

**Keywords:** ADHD, Cross-culture, Data linkage, Stimulant, Cohort study, Country-of-birth

## Abstract

**Background:**

To validate the association between country-of-birth and disparities in the stimulant use for ADHD among individuals in Western Australia.

**Methods:**

Using linked data, a population-based retrospective cohort of individuals admitted to hospital before age 25 years was followed through to identify having stimulants for ADHD in 2003–2007. Multivariate logistic and linear regressions were used to characterise associations between stimulants and country-of-birth, geographical remoteness and socioeconomic status.

**Results:**

Of 679,645 individuals, 14,122 (2.1%) had a record of having stimulants for ADHD. Of these, 205 (1.5%) were born in Africa, Asia, Middle-East or South America, while 13,664 (96.8%) were born in Australia/New Zealand, Europe or North America. Individuals with traditionally non-Anglophonic backgrounds were around one-half as likely to have stimulants as individuals with Anglophonic backgrounds (OR = 0.53, 95% CI 0.46–0.61, p < 0.001). Non-Anglophones were an average of 2.7 years older than Anglophones at onset of having stimulants. Individuals from remote and disadvantaged backgrounds had stimulants at younger ages than individuals living in metropolitan areas and with least disadvantage.

**Conclusions:**

The results highlight the importance of identifying factors underlying cultural differences in stimulant treatment for ADHD. Improving awareness of cultural variations may foster trust and rapport between patients and clinicians, and so better facilitate the appropriate and effective treatment of ADHD for each patient.

## Background

Attention deficit hyperactivity disorder (ADHD) is a commonly diagnosed chronic neurodevelopmental disorder in children and adolescents [1]. Psychostimulant medications are the most widely prescribed for ADHD treatment and the use of these medications has risen sharply in many parts of the world, particularly in European [2] and Western nations [3] over the last decade. In Australia, the stimulant prescribing rate for ADHD rose 72% between 2000 and 2011 [4], despite a significant community concern that ADHD is over-diagnosed and over-treated [5]. It is surprising that we have limited information on cultural variations in prescription stimulant in Australia, and if cultural attitudes towards ADHD diagnosis and treatment influence medication use. In a previous study, Ghosh et al. [6] reported about 83% lower stimulant use among ethnic minorities in Western Australia (WA) and attributed the disparities to parental country of birth differences. We examined differences in prescription stimulant between children and young adults born in traditionally Anglophonic and non-Anglophonic nations, in order to validate the earlier findings, but in this instance using the countries of birth of the children. Using a similar methodology, we hypothesized that an individual’s country of birth would result in variation in stimulant use for ADHD. A more general context for our research is the value we place on promoting ecologically sensitive medical practice, when empirically justified.

## Methods

### Data sources

**Fig. 1:**
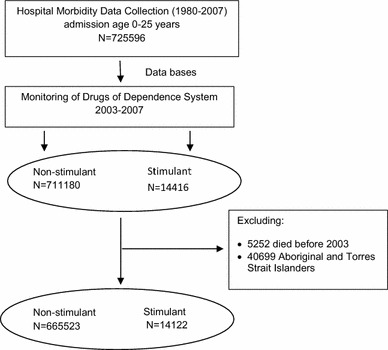
Study design, sample selection and exclusion criteria. Hospital Morbidity Data Collection was linked to Monitoring of Drugs of Dependence dataset to identify individuals who were admitted into hospital for the first time at age 0–25, and then to match with individuals who had prescription stimulant for ADHD. The final number of the study population was stimulant 14,122 and non-stimulant 665,523

We conducted a population-based retrospective cohort study using the WA Data Linkage System and linked three state-wide statutory health databases: the Hospital Morbidity Data Collection (HMDC) for inpatient separation, Monitoring of Drugs of Dependence System (MODDS) for stimulant records, and the Deaths Register [7]. The WA linkage system is well-known for high sensitivity (95–99%) and specificity (98–99%) [7]. The cohort included all individuals, ages 0–25 years, who were admitted into a hospital for any reason during 1980–2007, and followed through time to identify those who had a prescription stimulant for ADHD between 2003 and 2007 (Fig. 1).

The HMDC is a core dataset routinely linked by the WA Data Linkage Branch to other administrative health data and is one of the largest data collections managed by the WA Department of Health (WADoH) [8]. In 2012–2013, the HMDC comprised over 21 million electronic records of all public and private hospital stays in WA since 1970 [9]. The HMDC cohort was linked to the MODDS records to identify patients with an ADHD diagnosis and dispensed stimulant prescription. Under a WA Stimulant Regulatory Scheme enforced from 2003, public and private specialist medical practitioners in paediatrics, psychiatry, neurology and rehabilitation medicine were required by statute to obtain a stimulant prescriber number from the WADoH to be authorised to prescribe stimulant medications [10, 11]. The authorised prescribers were required to notify the WADoH of every commencement, alteration and termination of treatment with stimulant medications in all community, outpatient and inpatient settings. Similarly, it is a statutory requirement for all WA pharmacies to forward all information related to the dispensing of the stimulants to the WADoH to be stored in the MODDS [12]. Under the regulations, stimulant medications could only be prescribed for the treatment of ADHD, brain damage, depression or narcolepsy [10]. For the purpose of this study, data were collected where stimulants were prescribed for ADHD treatment only. ADHD could be diagnosed according to either the International Classification of Diseases, 10th revision (ICD-10), or the Diagnostic and Statistical Manual of Mental Disorders, fourth edition (DSM-IV) [11]. In Australia, short-acting dexamphetamine and long-acting methylphenidate are prescribed for ADHD treatment with stimulants, and considered cost-effective interventions for ADHD, since these are subsidized by the Government under the Pharmaceutical Benefits Scheme [11, 13].

WA is a state occupying the western third of the Australian continent with an estimated population of 2.5 million, around 11% of the national total in 2013 [14]. It was impossible to use census data to obtain a precise estimate of what proportion of the WA population aged 0–25 years were hospitalised at least once during 1980–2007. However, based on the relevant birth years of people resident in 2007, our approximate estimate was in the order of 40%.

### Variables and measurements

Geographical remoteness was scored according to the Accessibility/Remoteness Index of Australia of the Australian Census, using whichever of the 1996, 2001 or 2006 indices were closest to the year of cohort entry, and was grouped into three levels: metropolitan, rural and remote [15]. The index of relative socio-economic disadvantage from the socio-economic indexes for areas was used to categorise the study population into five levels of socioeconomic disadvantage ranging from most disadvantaged to least disadvantaged [15].

Individuals’ countries of birth were grouped under eight major geographical regions of the world according to eHRAF [16] databases—an internationally recognised anthropological databases facilitating study of human culture, society and behaviour. The eight groups were: Asia, Europe, Africa, North-America, Middle-East, Oceania (including Australia and New Zealand), Central-America and the Caribbean, and South America. The method used to classify country of birth are documented in detail in Ghosh et al. [6] paper where a pattern of reduced stimulant use was identified among individuals who were born in Africa, Asia, Middle-East or South America compared with those born in Australia/New Zealand, Europe or North America. As a result, we aggregated country of birth into Higher Propensity National Origin (HPNO), including Australia/New Zealand, Europe and North America, and Lower Propensity National Origin (LPNO) status for rest of the countries. For the purposes of the current study we focused our comparison on country of birth based on an individual’s HPNO and LPNO status.

### Statistical analysis

The outcome measure was at least one prescription stimulant for ADHD dispensed during 2003–2007. Descriptive statistics were performed for all study variables, including means and standard deviations obtained for continuous variables, and frequencies and percentages for categorical variables. Univariate and multivariate logistic regression models were used to examine factors associated with stimulant use. Individuals’ age for initial prescription stimulants between 2003 and 2007 were compared using multiple linear regression. Missing values where information was unknown for each variable were treated as a separate ‘unknown’ exposure category so that all subjects were included in the analyses. Individuals who died prior to 2003 were excluded from the study sample. Individuals identified themselves as Aboriginals were also excluded from the analysis. This exclusion followed the methods employed in a previous study due to likely cultural differences in the understanding of ADHD and attitudes towards medication between Aboriginals and non-Aboriginals [6].

## Results

Of the 679,645 individuals admitted to hospital for the first time by the age 25 years, 14,122 (2.1%) received a prescription stimulant for ADHD treatment during 2003–2007. The characteristics of the cohort are shown in Table 1. Of individuals who received stimulants, the majority of them (n = 13,664, 96.8%) were born in Australia/New Zealand, North America or Europe, with fewer (n = 205, 1.5%) born in Africa, Asia, Middle-East or South America. Nearly 2% (n = 253) did not have country of birth information. Individuals in rural and remote parts of WA comprised 17% (n = 2401), compared with 70% (n = 9869) who resided in the metropolitan area. Another 13% had missing residential information. Nearly one half (n = 6650, 47%) came from the least socioeconomically disadvantaged group, whilst 7% (n = 1002) belonged to the most disadvantaged group. There were 3.3 times more males receiving stimulant than females (76.7 vs. 23.3%).

**Table 1:** Characteristics of the study population according to stimulant medication use for ADHD

Characteristics	No stimulant used (%)	Stimulant used for ADHD (%)
Participants	665,523	14,122
Sex		
Male	340,226 (51.1)	10,834 (76.7)
Female	325,295 (48.9)	3288 (23.3)
Unknown	2 (0.01)	
Age at initial stimulant use between 2003 and 2007		
0–4 years	–	108 (0.8%)
5–12	–	7065 (50.0)
13–17	–	3941 (27.9)
≥18	–	3008 (21.3)
HPNO/LPNO status		
HPNO	635,914 (95.6)	13,664 (96.8)
LPNO	18,656 (2.8)	205 (1.5)
Unknown	10,953 (1.6)	253 (1.8)
Geographical remoteness		
Metropolitan	421,764 (63.4)	9869 (69.9)
Rural	111,860 (16.8)	2130 (15.1)
Remote	26,302 (4.0)	271 (1.9)
Unknown	105,597 (15.9)	1852 (13.1)
Social disadvantage		
Least disadvantaged	293,172 (44.1)	6650 (47.1)
Less disadvantaged	141,043 (21.2)	2916 (20.6)
Little disadvantaged	54,297 (8.2)	1198 (8.5)
More disadvantaged	26,630 (4.0)	553 (3.9)
Most disadvantaged	47,521 (7.1)	1002 (7.1)
Unknown	102,860 (15.5)	1803 (12.8)

HPNO includes Australia/New Zealand, Europe and North America, LPNO includes Africa, Asia, Middle East and South America

Both univariate and multivariate models indicated that individuals with LPNO backgrounds were approximately half as likely to receive stimulants compared with those with HPNO backgrounds (OR 0.53, 95% CI 0.46–0.61, p < 0.001) (Table 2). Females were 69% less likely to receive stimulants than males (OR 0.31, 95% CI 0.30–0.32, p < 0.001). The odds of receiving a stimulant were significantly lower in those living in rural (OR 0.80, 95% CI 0.77–0.84) and remote areas (OR 0.43, 95% CI 0.38–0.48) than in the metropolitan areas. At univariate level the odds for having stimulants was 1.8 times greater (OR 1.08, 95% CI 1.01–1.15) in the less disadvantage group than in the least disadvantaged, but the trend did not continue with greater levels of disadvantage. None of the examined disadvantaged group was a strong determinant of stimulant use after adjustment.

**Table 2:** Odds ratios for the association of at least one record of stimulant treatment for ADHD with cultural and demographic factors

Risk factors	Univariable		Multivariable^a^	
	OR (95% CI)	P value	OR (95% CI)	P value
HPNO/LPNO status				
HPNO^b^	1.00		1.00	
LPNO	0.51 (0.45–0.59)	<0.001	0.53 (0.46–0.61)	<0.001
Sex				
Male^b^	1.00		1.00	
Female	0.32 (0.31–0.33)	<0.001	0.31 (0.30–0.32)	<0.001
Geographical remoteness				
Metropolitan^b^	1.00		1.00	
Rural	0.81 (0.78–0.85)	<0.001	0.80 (0.77–0.84)	<0.001
Remote	0.44 (0.39–0.50)	<0.001	0.43 (0.38–0.48)	<0.001
Social disadvantage				
Least disadvantaged^b^	1.00		1.00	
Less disadvantaged	1.08 (1.01–1.15)	<0.01	1.02 (0.96–1.10)	0.52
Little disadvantaged	0.98 (0.91–1.05)	0.60	0.97 (0.90–1.04)	0.33
More disadvantaged	1.05 (0.96–1.14)	0.30	1.02 (0.94–1.12)	0.60
Most disadvantaged	0.99 (0.89–1.10)	0.78	0.96 (0.86–1.06)	0.41

^a^All parameters were included in this model so as to adjust each result for potential confounding by other covariates

^b^ Reference category

The mean age at onset of stimulants for those with a LPNO background was nearly three years older than in those with a HPNO background (14.98 vs. 12.27 years after adjustment, p < 0.001) (Table 3). Males received stimulants at an average age of 12.78 years, about two years younger than females at 14.47 years. Individuals from rural and remote areas were also about one year younger than those from the metropolitan area. Similarly those from the least disadvantaged group were between 1.28 and 1.63 years older at the time of stimulant treatment compared with other socioeconomic groups.

**Table 3:** Mean age in years in those receiving a stimulant medication for ADHD according to cultural and demographic factors

Predictors	Univariable			Multivariable^a^		
	Mean age	Mean difference (95% CI)	p value	Mean age	Mean difference (95% CI)	p value
HPNO/LPNO status						
HPNO^b^	13.10			12.27		
LPNO	16.08	2.99 (2.29, 3.68)	<0.001	14.98	2.72 (2.00, 3.431)	<0.001
Sex						
Male^b^	12.82			12.78		
Female	14.32	1.50 (1.30, 1.70)	<0.001	14.47	1.70 (1.48, 1.90)	<0.001
Geographical remoteness						
Metropolitan^b^	13.13			14.23		
Rural	12.07	−1.06 (−1.30, −0.83)	<0.001	13.63	−0.60 (−0.84, −0.36)	<0.001
Remote	11.50	−1.63 (−2.24, −1.02)	<0.001	13.01	−1.21 (−1.82, −0.61)	<0.001
Social disadvantage						
Least disadvantaged^b^	13.65			14.83		
Less disadvantaged	12.18	−1.47 (−1.70, −1.26)	<0.001	13.54	−1.28 (−1.50, −1.07)	<0.001
Little disadvantaged	12.03	−1.62 (−1.93, −1.31)	<0.001	13.38	−1.45 (−1.76, −1.14)	<0.001
More disadvantaged	11.82	−1.83 (−2.27, −1.40)	<0.001	13.17	−1.65 (−2.09, −1.22)	<0.001
Most disadvantaged	11.85	−1.80 (−2.14, −1.47)	<0.001	13.20	−1.63 (−1.96, −1.29)	<0.001

^a^ All parameters were included in this model so as to adjust each result for potential confounding by other covariates

^b^ Reference category

## Discussion

Our study found that prescription stimulants in WA varied significantly according to an individual’s country of birth. In particular, the odds of having stimulants in those born in Africa, Asia, Middle-East or South America were around one-half, and the mean age was 2.7 years older than in those born in Australia, Europe or North America. These findings are consistent with those documented in the earlier Ghosh et al. [6] study where a cohort of people born in WA between 1980 and 2007 was followed through time to identify those who had a stimulant record between 2003 and 2007 for ADHD treatment. That study employed a whole-population Australian birth cohort and used parental countries of birth as a proxy for ethnic groups, and so was not reliant on hospital admission for membership. The current study, on the other hand, had the advantage of including individuals born overseas, ascertained ethnic group using the individuals’ country of birth (not the parents). Our present results provide a validation of the earlier conclusions. The two different studies used cohorts constructed and measured in different ways, and were thus affected by different potential sources of error concerning external validity; yet the two studies have yielded similar results.

There were several limitations of the method used in this study. First, the individual’s country of birth variable was used as a surrogate given the absence of more direct ethnicity information. Groupings based on country of birth limited the capacity to detect differences between ethnicities and regional variations within countries to some extent, although it is generally agreed that country of birth plays a role in influencing individuals’ beliefs and attitudes. Second, detailed immigration information was unavailable and, therefore, we could not evaluate the association between stimulant use and refugee status or skilled migration. Third, our datasets did not allow us to identify anyone diagnosed with ADHD, yet not treated with stimulants. Fourth, as the stimulant data were only available from 2003, starting from initiation of the Stimulant Regulatory Scheme in WA, the first record in the stimulant dataset might not have been the first stimulant record in an individual’s life. In the absence of information prior to 2003, we were unable to observe the duration of stimulant treatment or dose information and, therefore, possible progression of ADHD symptoms in an individual’s life. Findings were further limited by the fact that records of dispensed prescription stimulant may not always determine the actual pattern of stimulant use for ADHD treatment, especially because there is a high prevalence of diversion and misuse of pharmaceutical stimulants among the adolescent and young adult student populations with ADHD [17]. Nevertheless, our study contributes to emerging evidence of the existence of ethnic differences in stimulant use, as we attempt to understand how ADHD behaviour is conceptualised in cross-cultural settings.

The strengths of this study compared with previous published studies were the use of whole-population linked data on a study population of nearly 700,000; a study cohort representing approximately 40% of the WA population aged 0–25 years between 1980 and 2007; and use of a comparison group of people with no records of stimulant use for ADHD. Whilst the representativeness of our cohort and the purity of exposure contract were far from perfect, they were superior to what can be achieved in a clinic-based research setting where subjects are typically highly selected and their treatments obtained outside unreliably recorded.

Cultural attitude towards ADHD behaviour, and resistance to accept a biomedical cause of ADHD and medication treatment are the prime reasons for a reduced likelihood of prescription stimulant and delayed onset of stimulant use among LPNO groups. Individuals’ perceptions of normal and pathological behaviour are largely determined by cultural beliefs which influence their care-seeking behaviour [18]. For example, ADHD behaviour was viewed by school teachers in India as childhood transition likely to improve as the child grew older [19]. The researcher reported that the ADHD behaviour was perceived by the teachers as a positive trait of a child with higher physical energy levels and cognitive abilities. This cultural complexity in understanding ADHD behaviour in Indian society was reported to lead to a 6-year gap between the noticing ADHD symptom and making a diagnosis [20]. Similarly, culture-specific differences in attitudes towards symptoms of ADHD were reported in Iranian culture, where ADHD was viewed as signs of normal child development and independence leading to at least a two-year delay in treatment [21]. Perceptual differences were also observed among Moroccan, Turkish and Surname immigrants in the Netherlands, with a higher treatment threshold for ADHD behaviour, resulting in a lower number of prescription medications in those immigrant children than in native Dutch children [22]. Willingness to prefer medication treatment for ADHD behaviour is a pivotal cultural question that needs to be investigated.

Lower rates of medication treatment and delay in treatment were documented among Latino adolescents and youth in Venezuela largely due to a reluctance to accept medication treatment [23]. Research suggested that cultural beliefs about the aetiology of ADHD influenced African–American and Latino communities to pursue alternative forms of treatment or decide not to pursue treatment at all [24]. Even when they accepted biomedical causes of ADHD, behavioural intervention was their preferred method of treatment rather than stimulant medication [25]. Substantial ethnic disparities continue to exist for stimulant treatment of ADHD and other mental and behavioural problems in Netherlands [26], Spain [27] and Sweden [28].

Our findings also revealed that males were 69% more likely to receive stimulant than females possibly due to gender variation in ADHD manifestations, where boys exhibit more hyperactivity than girls, who display mostly inattentiveness [29]. As ADHD is considered a disorder of academic performance, excitability may cause disruption in a classroom situation, resulting in frequent diagnosis referrals and subsequent treatment in boys sometime even without valid cause [30]. While a high stimulant prevalence in boys is widely reported, a growing number of girls are being medicated for ADHD, leading to a declining male:female ratio in Australia [31].

We found individuals living in the metropolitan areas were more likely to have prescription stimulants. Studies have examined differential healthcare access, availability of physicians and ready access to healthcare services in major cities as factors influencing regional variation in stimulant treatment [32]. Differences in beliefs and values about child behaviour and medical treatment, and the willingness to accept stimulant treatment may also vary geographically, contributing to a regional disparity [33]. The older age at commencement of stimulants in the metropolitan area than in rural areas in our results is a more difficult finding to explain. It may reflect a difference between metro and rural in the use of medication to improve academic outcomes, more so than to alleviate disruptive behaviours [34] as those affected were aged more into their teenage years. A positive correlation between lower socioeconomic status and higher psychostimulant treatment has been documented previously in national and international studies [35, 36]. Due to large socioeconomic discrepancies in stimulant use, some studies have uncovered concerns that psychosocial issues associated with socioeconomic disadvantage may be misattributed as symptoms of ADHD in children, leading to suggestions that medicalising behaviour, that might have been considered normal in the past, is a popular global phenomenon [37, 38].

## Conclusions

Using a different method, this study validated cultural differences in stimulant treatment for ADHD reported in previous research, and identified significant country of birth variations, as well as gender, regional and socioeconomic disparities in stimulant use for ADHD in WA. Individuals born in Africa, Asia, Middle-East or South-America were less likely to have stimulant treatment than individuals born in Australia/New Zealand, Europe or North America. A greater likelihood of stimulant treatment among boys, individuals living in metropolitan areas, and living with socioeconomic disadvantage was also observed. The findings highlight the need for tailoring ADHD diagnosis, treatments and service delivery appropriately to children and adolescents from diverse cultures.

## Abbreviations

ADHD: attention deficit hyperactivity disorder
HMDC: Hospital Morbidity Data Collection
HPNO: Higher Propensity National Origin
LPNO: Lower Propensity National Origin
MODDS: Monitoring of Drugs of Dependence System
OR: odds ratios
WA: Western Australia
WADoH: Western Australia Department of Health
